# Lipid abnormality in diabetic kidney disease and potential treatment advancements

**DOI:** 10.3389/fendo.2025.1503711

**Published:** 2025-03-18

**Authors:** Qian Ming Tu, Hui Min Jin, Xiu Hong Yang

**Affiliations:** ^1^ Department of General Medicine, Shanghai Putuo District Changzheng Town Community Health Service Center, Shanghai, China; ^2^ Department of Internal Medicine, Shanghai Dong Ji Fresenius Hemodialysis Center, Shanghai, China; ^3^ Department of Nephrology, the People’s Hospital of Wenshan Prefecture, Yunnan, China; ^4^ Division of Nephrology, Shanghai Pudong Hospital, Fudan University, Pudong Medical Center, Shanghai, China; ^5^ Department of Nephrology, Huadong Hospital, Fudan University, Shanghai, China

**Keywords:** lipid, diabetic kidney disease (DKD), triglyceride (TG), dyslipidemia, diabetes complications

## Abstract

Numerous studies have shown that dyslipidemia increases the risk of atherosclerotic cardiovascular disease (ASCVD) and significantly impacts the occurrence and progression of diabetic kidney disease (DKD). Early interventions for lipid metabolism disorders in DKD may improve renal function. This article reviews the clinical characteristics of dyslipidemia, mechanisms of lipid-induced renal injury, and advances in lipid-lowering therapy in DKD. We searched PubMed, Web of Science, and EMBASE to identify relevant articles, using keywords such as “diabetic kidney disease”, “diabetic nephropathy”, “diabetes”, “dyslipidemia”, “kidney”, “cardiovascular disease”, and “lipid therapy”. High triglyceride (TG) and low high-density lipoprotein (HDL) are associated with increased risks of albuminuria and estimated glomerular filtration rate (eGFR) decline. Abnormal lipid metabolism may damage glomerular podocytes and renal tubular epithelial cells via ectopic lipid deposition, eventually impairing glomerular filtration function and increasing urinary albumin excretion. Lipid-lowering therapies can ameliorate lipid accumulation, downregulate inflammatory mediator expressions, and alleviate renal fibrosis. Fibrate and statin applications exhibit beneficial effects, reducing albuminuria and slowing eGFR decline in early DKD. However, the long-term effects of statins and fibrates on renal outcomes remain controversial. Pro-protein convertase subtilisin/kexin 9 (PCSK9)-targeted interventions have minimal side effects on the kidneys and seem effective in reducing inflammation and improving renal impairment compared with statins and fibrates. In addition, LDL apheresis (LDL-A) and double filtration plasmapheresis (DFPP) are promising clinical applications in diabetic patients with severe hypercholesterolemia or lipid-lowering drug intolerance.

## 1 Introduction

Diabetic kidney disease (DKD) is clinically diagnosed in patients exhibiting a reduction in their estimated glomerular filtration rate (eGFR) to <60 ml/min/1.73m^2^, persistent elevation of albuminuria (Urine Albumin-to-Creatinine Ratio [UACR] ≥ 30 mg/g), or both. The main pathological changes in DKD include glomerular capillary basement membrane thickening, mesangial cell proliferation, glomerulosclerosis, and tubulointerstitial fibrosis (1). It is the main cause of chronic kidney disease (CKD) and end-stage renal disease (ESRD). From 2017 to 2018, the overall prevalence of CKD among diabetic patients in a central Chinese urban population was 48% (2). In United States of America, approximately 30% to 40% of individuals with diabetes develop DKD during their lifetime (3).

Numerous studies have shown that dyslipidemia increases the risk of atherosclerotic cardiovascular disease (ASCVD) and significantly impacts the occurrence and progression of diabetic nephropathy (DN). The risk of cardiovascular disease, cardiovascular death, and all-cause mortality markedly increased in type 2 diabetes mellitus (T2DM) patients with DKD complications (4, 5). Moreover, literatures indicate that DKD has been shown to be an independent risk factor for cardiovascular disease. Additionally, it suggests that the severity of renal impairment correlates with an increased risk of cardiovascular complications and other associated risks (6–9). Farah, RI et al. found that half of the T2DM patients had DKD, and high triglyceride (TG) and low high-density lipoprotein (HDL) were outstanding features in those patients (10). In 2021, the Chinese Cardiometabolic Diseases and Cancer Cohort (4C) study suggested that a high TG level (TG ≥ 1.70 mmol/L) was an independent risk factor for DKD development in subjects with new-onset T2DM (11). Furthermore, postprandial TG variability is significantly related to eGFR decline and microalbuminuria incidence in patients with T2DM (12). Early intervention for lipid metabolism disorders in DKD may alleviate renal damage. This article reviews the clinical characteristics of dyslipidemia, mechanisms of lipid-induced renal injury, and advances in lipid-lowering therapy in DKD.

## 2 Clinical characteristics of dyslipidemia in DKD

### 2.1 Dyslipidemia in different stages of DKD

Dyslipidemia is highly prevalent among subjects with DKD and is primarily characterized by elevated TG, reduced HDL-C, and normal or mildly elevated low-density lipoprotein cholesterol (LDL-C) (13). As renal functions deteriorate, TG levels gradually increase, whereas HDL-C levels further decrease (14). In the early stages of DKD, dyslipidemia is primarily caused by elevated TG. After entering the stage of massive albuminuria, TG, LDL-C, and small and dense low-density lipoprotein cholesterol (sdLDL-C) increase substantially, while HDL-C decreases significantly. At the terminal stage of DKD, lipid metabolism disorders are further aggravated, with an increase in very-low-density lipoprotein cholesterol (VLDL-C) (15, 16).

### 2.2 Dyslipidemia is associated with increased risks of albuminuria and eGFR decline

Recent studies have revealed that lipid abnormalities strongly affect the long-term deterioration of renal function in diabetes. A large-scale global case–control study indicated that the risk of developing DKD increased by 23% for every 0.5 mmol/L increase in TG and decreased by 14% for every 0.2 mmol/L increase in HDL-C (17). In China, a 3-year follow-up study involving 283 subjects with new-onset T2DM suggested that inadequate TG control was associated with an early decrease in eGFR; effective TG control could delay the decline of kidney function in the early stages of DKD (18). Another real-world Italian cohort containing 45000 individuals with low-to-moderate cardiovascular risk demonstrated that subjects with high TG (150-500 mg/dL) had an increased risk of eGFR reduction or ESRD occurrence (composite endpoint) by 48% compared with normal TG subjects (<150 mg/dL) (19). Moreover, each 50 mg/dL increase in TG levels resulted in a statistically higher risk of eGFR reduction (OR:1.062, 95%CI 1.039-1.086) and ESRD (OR:1.174, 95%CI 1.070-1.289). Russo et al. found that TG ≥ 150 mg/dL increased the risk of the following: low eGFR (< 60 mL/min/1.73 m^2^) by 26%, >30% eGFR reduction by 29%, and albuminuria by 19%. They also found that HDL-C levels of <40 mg/dL in men and <50 mg/dL in women were associated with a 27% higher risk of low eGFR and a 28% risk of a >30% eGFR reduction, with a 24% higher risk of developing albuminuria (14). Furthermore, plasma TG levels independently predict incident albuminuria and the progression of coronary artery calcification in adults with type 1 diabetes (20).

### 2.3 Predictive value of TG/HDL-C ratio in the progression of DKD

The TG/HDL-C ratio may have a better predictive value for DKD development compared to a single lipid parameter. A cross-sectional survey based on six community health service centers in Shanghai indicated that the possibility of developing DKD in patients with T2DM significantly increased when the TG/HDL-C ratio was >2 (21). The TG/HDL-C ratio correlated positively with UACR and negatively with eGFR, which was a critical risk factor for severe DKD (UACR ≥30 mg/g and eGFR <60 mL/min/1.73 m^2^) in patients with T2DM and advanced diabetic retinopathy (22). A higher TG/HDL-C ratio was also statistically associated with higher incidences of cardiovascular disease among patients with biopsy-proven diabetic nephropathy (23).

### 2.4 Mechanisms of lipid-induced renal dysfunction

Among diabetic patients, insulin resistance (IR) motivates hormone-sensitive triglyceride lipase (HSL), stimulating the release of free fatty acids (FFA) from peripheral adipose tissue. High circulating FFA levels promote the liver to synthesize triglycerides, causing an increase in plasma TG concentration, inversely reducing HDL-C synthesis via cholesterol ester transfer protein (CETP) (24). The disturbances of lipid metabolism bring about ectopic lipid deposition in non-adipose tissues such as the liver, kidneys, heart, and pancreas.

In contrast to normal kidney tissue, the glomeruli and tubulointerstitial of diabetic nephropathy contain abundant TG-rich lipid droplets (25). TG accumulations activate monocytes to release inflammatory mediators, which stimulate transforming growth factor-β (TGF-β) secretion and reactive oxygen species (ROS) production, thus resulting in renal cell injury and matrix proliferation (18). Furthermore, podocytes are particularly sensitive to lipid accumulation, which has recently been recognized as a crucial pathological process in the progression of proteinuric kidney diseases, such as diabetic kidney disease. Excessive lipid deposition in podocytes disrupts cellular homeostasis, leading to lipotoxicity. This can induce mitochondrial oxidative stress, impair energy metabolism, and promote ROS overproduction, further damaging mitochondrial DNA and disrupting ATP production. Ultimately, this triggers podocyte apoptosis and detachment from the glomerular basement membrane. Additionally, lipid overload can alter the actin cytoskeleton, causing dysregulation of key cytoskeletal regulators such as RhoA and Rac1, which leads to foot process effacement and increased glomerular permeability. Lipotoxicity has also been shown to impair insulin signaling in podocytes by inhibiting the PI3K/Akt pathway, exacerbating insulin resistance and further compromising podocyte survival. These combined effects weaken the integrity of the glomerular filtration barrier, facilitating albumin leakage and proteinuria, which are hallmarks of diabetic kidney disease progression (25–27).

Recent studies have highlighted the morphological alterations in podocytes due to lipid accumulation. Excessive lipid deposition leads to the formation of intracellular lipid droplets, which are visible under electron microscopy. These droplets are often accompanied by structural changes in the podocyte foot processes, including effacement and fusion, which are critical features in the progression of diabetic kidney disease. Lipid-induced injury to podocytes has also been linked to significant changes in the morphology of the glomerular filtration barrier, with thickening of the glomerular basement membrane and enlargement of the filtration slits. These morphological changes contribute to disruption in podocyte function, exacerbating glomerular hyperpermeability and the progression of proteinuria (27, 28). Moreover, increased lipolysis of TG-rich lipoproteins (TRLs) begets sd-LDL-C formations, a more atherogenic lipoprotein than large-buoyant LDL particles. Easily deposited in the renal basement membrane, sd-LDL-C stimulates extracellular matrix proliferation and undergoes oxidative modification, promoting foam cell formation and lipid sedimentation in the vascular wall (16).

**Figure 1:**
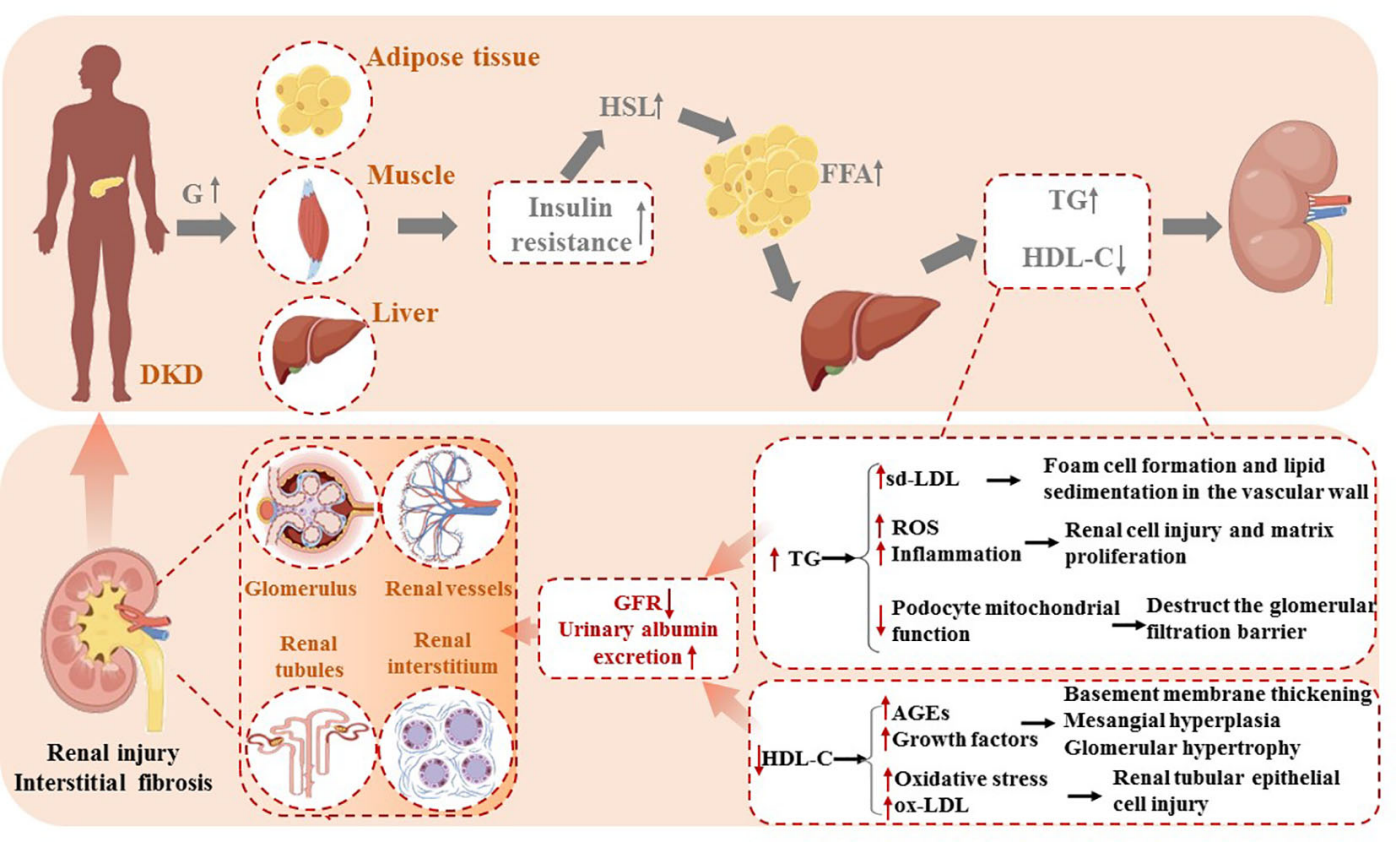
Mechanisms of lipid-induced renal dysfunction.

Involving low levels of HDL-C in the pathogenesis of DKD may be linked to the impaired antioxidant capacity of HDL-C. Under normal circumstances, the monocyte–macrophage system eliminates advanced glycation end products (AGEs) as markers of aging via endocytosis and secretes cytokines to stimulate extracellular matrix synthesis. However, the impaired antioxidant capacity of HDL particles contributes to elevated AGE content and excessive secretion of growth factors, eventually leading to vascular basement membrane thickening, mesangial hyperplasia, and glomerular hypertrophy (13). Oxidative stress (OS) is present even in the early stages of DKD due to the imbalance between pro- and antioxidants. OS facilitates the oxidative conversion of LDL to oxidized LDL (ox-LDL) within the arterial wall. ox-LDL can directly damage the renal tubular epithelial cells via a mechanism similar to the deleterious effects of lipid peroxides on the vascular endothelium (29). These factors ultimately cause renal function damage, leading to a decreased glomerular filtration rate and increased urinary albumin excretion (**Figure 1**).

## 3 Advances in treating dyslipidemia in DKD

### 3.1 Statins

Statins, 3-hydroxy-3-methylglutaryl-coenzyme A (HMG-CoA) reductase inhibitors, significantly reduce serum LDL-C levels, mildly reduce serum TG levels, and increase HDL-C levels. Many clinical studies have confirmed that statins for diabetics can effectively reduce lipids and prevent cardiovascular events. Li et al. found that atorvastatin could alleviate renal function damage, reduce serum inflammatory factor levels, and improve hemorheology in patients with DKD (30). Findings from two meta-analyses consistently indicated that statins have beneficial effects on reducing albuminuria in DKD (31, 32). The efficacy of statins on renal function was dependent on time, and the decrease in albuminuria was significant during the 1-to-3-year period of statin therapy (31). Moreover, another meta-analysis of 3,426 patients with early diabetic nephropathy concluded that statins could increase eGFR, reduce serum creatinine (Scr), and decrease C-reactive protein (CRP) level, thus reducing the inflammatory response and protecting the kidney (33).

However, the renoprotective effects of statins on diabetic patients are still controversial. A retrospective cohort study of patients with T2DM in Japan showed a higher risk of a ≥30% decrease in eGFR in the lipophilic statin group (atorvastatin and pitavastatin) than in the controls (HR = 2.15, p = 0.049); the outcome in the hydrophilic statin group (pravastatin and rosuvastatin) was not different (HR = 1.08, p = 0.827). The hazard ratio of the lipophilic and hydrophilic statin group (vs. controls) for albuminuria progression was 1.31 (p = 0.552) and 0.69 (p = 0.367) (34). Statins do not reduce the risk of progression of eGFR and albuminuria. Lipophilic statins might have potentially harmful effects on kidney function. Recently, Huang et al. reported that the insulin-regulated phosphatidylinositol 3-kinase (PI3K)/protein kinase B (PKB)/mammalian target of rapamycin (mTOR) signaling pathway was activated in db/db mice with the long-term administration of atorvastatin or rosuvastatin, which may increase insulin resistance, interfere with lipid metabolism, and lead to inflammation and fibrosis, ultimately exacerbating diabetic nephropathy progression (35). However, Huang stressed that statins in combination with metformin could improve the negative effects of statins on the kidneys. Lipophilic statins, such as simvastatin and lovastatin, can penetrate cell membranes, particularly in muscle and kidney tissues, which may exacerbate insulin resistance and worsen kidney function. Several studies have shown that lipophilic statins may increase fasting blood glucose levels, thus aggravating insulin resistance (35, 36). Additionally, these statins may affect renal tubular function, leading to interstitial fibrosis, which accelerates the progression of diabetic nephropathy (37). As most clinical studies have a relatively short follow-up time, it would need a long-term clinical application of larger sample sizes to make a more objective evaluation of statin therapy on the prognosis of DKD.

### 3.2 Fibrates

Fibrates, peroxidase proliferator-activated receptor α (PPAR-α) agonists, mainly reduce serum TG levels and increase HDL-C levels. A *post hoc* analysis of the ACCORD trial demonstrated that fenofibrate administration was associated with a lower rate of eGFR decline and with a lower incidence of both microalbuminuria (UACR ≥30 mg/g) and macroalbuminuria (UACR ≥300 mg/g) in T2DM (38). In the FIELD study, type 2 diabetic patients with eGFR ≥30 mL/min/1.73 m^2^ were randomly allocated to fenofibrate 200 mg daily (n = 4,895) or placebo (n = 4,900) for 5 years. Fenofibrate reduced urine albumin concentrations and UACR by 24% vs. 11%, with 14% less progression and 18% more albuminuria regression than placebo. The mean annual rates of eGFR loss were 1.19 vs. 2.03 mL/min/1.73 m^2^, respectively, for the fenofibrate group vs. placebo group. Overall, fenofibrate further reduced the total cardiovascular events than placebo (hazard ratio 0.89 [95% CI 0.80-0.99]). This benefit was not statistically different across eGFR subgroups (30-59, 60-89, and ≥90 mL/min/1.73 m^2^). ESRD rates were similar between treatment arms, without adverse safety signals of fenofibrate use in renal impairment (39, 40). Another national cohort study based on Taiwan’s National Health Insurance Research Database compared the outcomes among advanced CKD patients treated with fenofibrate, statins, a combination of both, and none of these. The fenofibrate and statin groups exhibited a lower risk of CV death than the non-user group, and the combined administration of fenofibrate and statins exhibited a lower risk of major adverse cardiac and cerebrovascular events. Moreover, fenofibrate further delayed the need for permanent dialysis (41). Fenofibrate mechanically ameliorates lipid accumulation and tubular cell apoptosis, accompanied by an increased activation of AMP-activated protein kinase (AMPK)/forkhead box protein A2 (FoxA2)/medium-chain acyl-CoA dehydrogenase (MCAD) pathway (42). MCAD may be a potential therapeutic target for DKD, and using fenofibrate as a treatment for DKD is worthy of further study.

**Figure 2:**
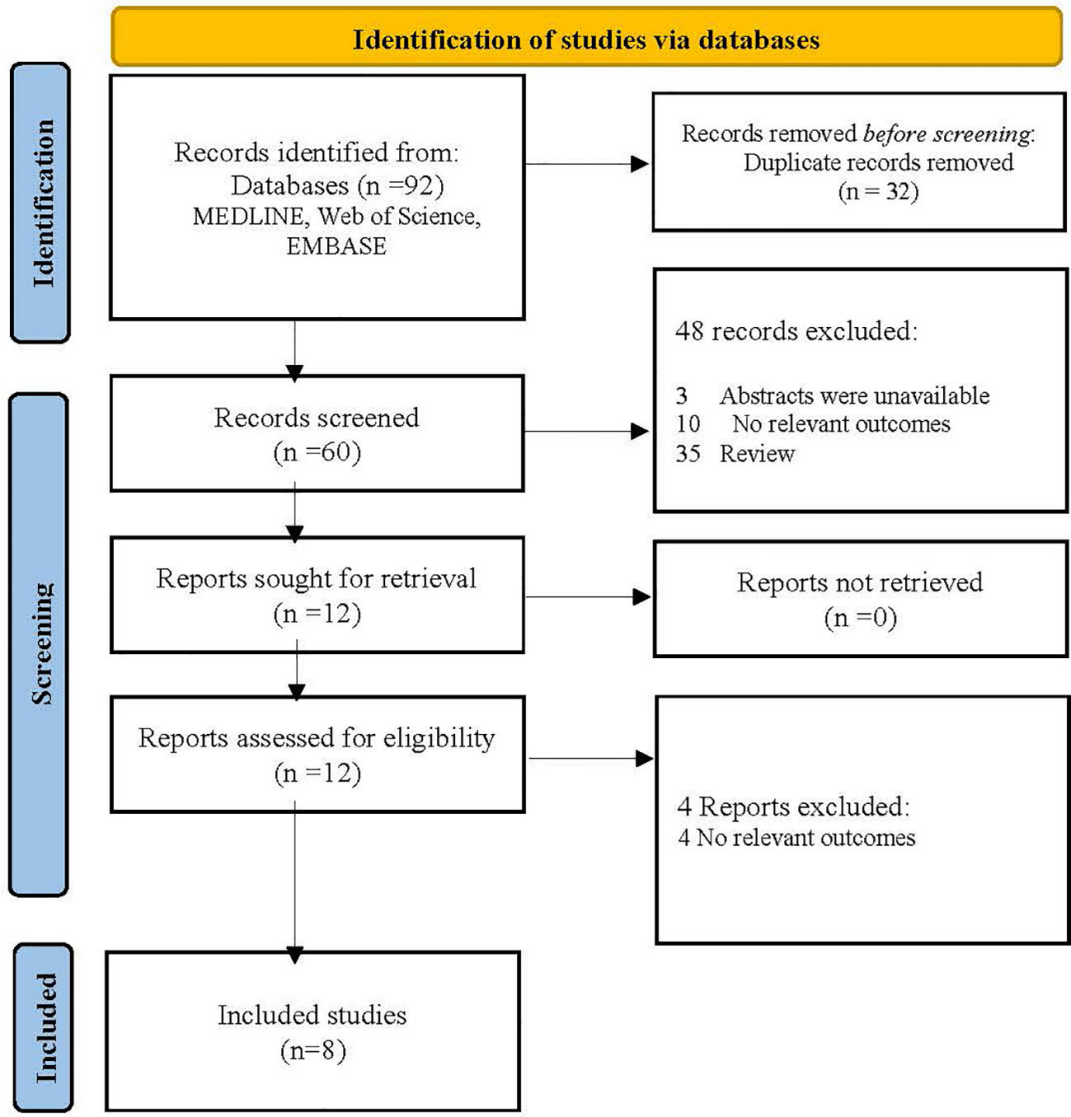
Flowchart of fibrate-related studies examining renal outcomes in the diabetic population.

Pemafibrate is a new selective PPAR-α modulator (SPPARM α). Conventional fibrates mainly eliminated via renal excretion may lead to an increase in serum creatinine (Scr), while pemafibrate is excreted into the bile with fewer side effects on the kidney. In the adenine-induced CKD mice, pemafibrate suppressed the Scr and blood urea nitrogen (BUN) levels and inhibited the upregulation of monocyte chemoattractant protein-1 (MCP-1), tumor necrosis factor-alpha (TNF-α), interleukin-1 beta (IL-1β), and interleukin-6 (IL-6) (43). A prospective observational study reported that switching from conventional fibrates to pemafibrate for 52 weeks improved eGFR but increased serum uric acid (UA) in T2DM patients (44). However, in the PROMINENT trial, pemafibrate did not reduce cardiovascular risk but was associated with a higher incidence of adverse renal events (CKD, DN, acute kidney injury, and proteinuria), despite lowering TG, VLDL-C, remnant cholesterol, and apolipoprotein C-III levels (45). The researchers indicated that pemafibrate-mediated reductions in triglyceride-rich lipoprotein remnants were converted into LDL particles as opposed to the liver removing them. Moreover, an ongoing open-label, randomized controlled clinical trial (PROFIT-CKD) aiming to evaluate the effectiveness of pemafibrate on urinary protein excretion will reveal new findings regarding pemafibrate’s impact on renal outcomes (46). The search strategies of databases, flowchart and basic characteristics of fibrate studies associated with renal outcomes in the diabetic population are shown in **Figure 2**, **Tables 1** and **2** (38, 39, 44, 45, 47–50).

**Table 1:** The search strategies of databases.

Databases	Search strategies
MEDLINE	Search (“diabetic kidney disease”[Mesh] OR “diabetic nephropathy”[Mesh] OR “diabetes”[Mesh]) AND (“fibrate”[Title/Abstract]) AND (“renal outcomes”[Title/Abstract] OR “kidney function”[Title/Abstract] OR “albuminuria”[Title/Abstract] OR “glomerular filtration rate”[Title/Abstract])
Web of Science	#1 TS=(fibrate) #2 TS=(diabetic kidney disease OR diabetic nephropathy OR diabetes) #3 TS=(renal outcomes OR kidney function OR albuminuria OR glomerular filtration rate) #3 AND #2 AND #1
Embase	#1 ‘fibrate’:ab,ti #2 ‘diabetic kidney disease’/exp OR ‘diabetic nephropathy’/exp OR ‘diabetes’:ab,ti #3 ‘renal outcomes’:ab,ti OR ‘kidney function’:ab,ti OR ‘albuminuria’:ab,ti OR ‘glomerular filtration rate’:ab,ti #3 AND #2 AND #1

**Table 2:** Characteristics of fibrate studies associated with renal outcomes in diabetic population.

Study	N	Age (years)	Country	Sex (male%)	Patients	Study type	Follow up	Fibrate group	Control group	Renal outcomes
Bruce R et al., 1996 (47)	24	58 ± 9	New Zealand	/	T2DM with mild renal impairment	RCT	6 months	Bezafibrate	Placebo	Decrease in albuminuria
Ansquer JC et al., 2005 (48)	314	56	France	75.5	T2DM	RCT	38 months	Fenofibrate	Placebo	Decrease in albuminuria
Davis TM et al., 2011 (39)	9795	50-75	Australia, New Zealand, and Finland	63	T2DM	RCT	5 years	Fenofibrate	Placebo	Decrease in albuminuria; Lower rate of eGFR decline;
Frazier R et al., 2018 (38)	5268	62.8 ± 6.6	United States	69.4	T2DM	RCT	4 years	Fenofibrate + simvastatin	Placebo + simvastatin	Decrease in albuminuria; Lower rate of eGFR decline;
Sun X et al., 2020 (49)	56	30-75	China	66	T2DM	RCT	180 days	Fenofibrate	No placebo	Decrease in albuminuria;
Yanai H et al., 2021 (50)	15	55.1 ± 12	Japan	53.3	T2DM	Retrospective study	12 months	Pemafibrate	Fenofibrate	Increase in eGFR
Kito K et al., 2022 (44)	504	61	Japan	64.5	T2DM	Prospective observational study	52 weeks	Pemafibrate	Conventional fibrates	Increase in eGFR
Das Pradhan A et al., 2022 (45)	10,497	58-70	Japan	72.5	T2DM	RCT	3.4 years	Pemafibrate	Placebo	Higher incidence of renal adverse events (CKD, DN, AKI, and proteinuria)

T2DM, type 2 diabetes mellitus; RCT, randomized controlled trial; eGFR, estimated glomerular filtration rate; CKD, chronic kidney disease; DN, diabetic nephropathy; AKI, acute kidney disease.

Although pemafibrate, a selective peroxisome proliferator-activated receptor alpha (PPARα) agonist, has demonstrated significant benefits in improving lipid profiles in several clinical trials, its long-term effects and safety, particularly in diabetic and kidney disease patients, remain controversial. Some studies suggest that pemafibrate may offer cardiovascular protection, while others indicate that its impact on kidney function is not yet fully understood and may potentially increase renal burden. Therefore, we recommend that the use of pemafibrate should be carefully assessed based on individual patient characteristics, especially renal function.

### 3.3 Proprotein convertase subtilisin/kexin type 9 inhibitors

PCSK9 inhibitors enhance the expression of low-density lipoprotein receptor (LDL-R) on the surface of hepatocytes to accelerate LDL catabolism and reduce plasma LDL-C levels. The main PCSK9 inhibitors currently on the market are PCSK9 monoclonal antibodies (mAbs), including evolocumab and alirocumab. Multiple randomized controlled trials claim that PCSK9 mAbs seem effective in reducing the risk of major cardiovascular events (MACE) and in improving the lipid profiles of subjects with diabetes and dyslipidemia (51). In high-fat diet/streptozotocin (HFD/STZ)-induced mice, PCSK9 mAbs inhibited the cyclic GMP-AMP synthase (cGAS)/stimulator of interferon genes (STING) signaling pathway associated with inflammation cascades and markedly reduced the Scr, BUN, and UACR indexes in DKD (52). Feng et al. found that the serum PCSK9 level was positively correlated with BUN, Scr, TG, and UACR but inversely correlated with eGFR. A logistic regression model revealed that PCSK9 was an independent risk factor for UACR ≥ 30 mg/g and eGFR<60 mL/min/1.73 m^2^ (53). Nevertheless, a cross-sectional study of 134 DKD patients suggested that the fibrate/statin combination therapy remained independently associated with higher PCSK9 levels. The biomarker potential of PCSK9 levels should be investigated to identify DKD patients who may benefit from anti-PCSK9 strategies (54).

Compared with statins, PCSK9 inhibitors have no significant side effects on the liver and kidney, presenting good safety and tolerability instead. However, PCSK9 mAbs must be injected every two weeks during the initial stages of the treatment. Inclisiran, a novel small interference RNA (siRNA) that inhibits PCSK9, is administered twice a year to achieve an effective and sustained reduction in LDL-C. Inclisiran maintenance therapy is a reasonable alternative to PCSK9 mAbs in patients who struggle with adherence to PCSK9 mAbs (55, 56). ORION-3, a 4-year open-label extension study of the placebo-controlled, phase 2 ORION-1 trial, suggested that LDL-C in the inclisiran-only arm was reduced by 47.5% at day 210, and the reduction was sustained over 1440 days. The 4-year average mean reduction in LDL-C was 44.2%, with reductions in PCSK9 ranging from 62.2% to 77.8% (57). Another patient-level pooled analysis of ORION-9, -10, and -11 revealed that a total of 141 MACEs were reported in the inclisiran group (n=1833) during the 18-month treatment, with 201 MACEs in the placebo group (n=1822). Inclisiran statistically reduced the risk of composite MACE (OR=0.74, 95%CI:0.58-0.94) in patients with heterozygous familial hypercholesterolemia, ASCVD, or an ASCVD risk equivalent for the maximally tolerated statin-therapy (58). This analysis offers early insights into the potential CV benefits of lowering LDL-C with inclisiran, while the effect of inclisiran on DKD patients awaits confirmation in future clinical studies.

Regarding PCSK9 inhibitors, although these agents have proven benefits in lowering LDL cholesterol and reducing cardiovascular events, their long-term safety, including any systemic side effects, remains a subject of ongoing research. Potential adverse effects such as injection site reactions, muscle pain, and a possible increased risk of diabetes need to be closely monitored in patients, especially those who may already be at higher risk due to their cardiovascular and metabolic conditions (57, 58).

### 3.4 Therapeutic plasmapheresis

For patients who have severe hypercholesterolemia or cannot tolerate lipid-lowering drugs, therapeutic plasmapheresis is highly recommended. Low-density lipoprotein apheresis (LDL-A) can selectively remove LDL, ox-LDL, and VLDL from plasma via powerful specific adsorption. In a Japanese multicenter prospective study (LICENSE), LDL apheresis was performed 6-12 times over 12 weeks in 40 diabetic patients with severe proteinuria and dyslipidemia. After 6 months, the proportion of cases with 30% or more reduced proteinuria in the LDL-A group was 25%. The overall survival and end-stage kidney disease-free survival rates were significantly higher in the LDL-A group than in historical controls (59). In addition, LDL apheresis may improve the mental and physical quality of life in patients with DKD (60).

Double filtration plasmapheresis (DFPP), a semi-selective blood purification modality with little or no albumin replacement, can filter plasma pathogenic substances such as lipids, autoantibodies, and inflammatory factors using component separators of different pore sizes. The second filter must be chosen for each patient according to the size of the lipoproteins. In diabetic patients with severe hypertriglyceridemia, the centrifuge-type blood cell separator might be more efficient than the hollow-fiber column for the plasma separation of triglyceride-rich lipoproteins (61). Recently, a clinical trial involving 45 adults with hyperlipidemia reported a significant reduction in all lipid profile components (TC, TG, HDL, and LDL) after DFPP treatment. As for uremic toxins and inflammation, CRP, UA, and alanine transaminase (ALT) levels decreased, which may be related to removing serum perfluorooctane sulfonate (PFOS) and improving renal functions (62). DFPP has a strong lipid-lowering activity and can regulate proteins related to apoptosis, lipid metabolism, and endoplasmic reticulum stress, reducing reactive oxygen species (ROS) and serum inflammatory factors release (63).

## 4 Conclusions

Abnormal lipid metabolism may damage glomerular podocytes and renal tubular epithelial cells via ectopic lipid deposition, eventually impairing glomerular filtration function and increasing urinary albumin excretion in DKD. Lipid-lowering therapies can ameliorate lipid accumulation, downregulate the expression of inflammatory mediators, and alleviate renal fibrosis. The application of fibrates and statins exhibits beneficial effects on reducing albuminuria progression and slowing eGFR decline in early DKD. However, the long-term effects of statins and fibrates on renal outcomes remain controversial and await confirmation in larger and longer sample trials. PCSK9-targeted interventions have minimal side effects on the kidney and seem effective in reducing inflammation and improving renal impairment compared with statins and fibrates. Incisiran, a novel PCSK9 siRNA, is administered twice a year to achieve a sustained and effective reduction in LDL-C, providing new therapeutic opportunities for DKD. Additionally, future studies are needed to further evaluate its long-term renal safety and potential effects on proteinuria to optimize lipid-lowering management strategies for DKD. Furthermore, LDL-A and DFPP are promising clinical applications in diabetic patients with severe hypercholesterolemia or lipid-lowering drug intolerance.

The traditional understanding of diabetic kidney disease (DKD) follows a progressive course characterized by glomerular hyperfiltration, microalbuminuria, overt proteinuria, and a subsequent decline in eGFR. However, recent evidence challenges this linear model, suggesting that DKD manifests in two distinct phenotypes: albuminuric DKD, characterized by persistent albuminuria, and non-albuminuric DKD, where renal impairment (eGFR < 60 ml/min/1.73m²) occurs without significant albuminuria (64). These two phenotypes may have different pathophysiological mechanisms, with non-albuminuric DKD more closely associated with tubulointerstitial damage, vascular dysfunction, and metabolic abnormalities, rather than glomerular injury alone. Non-albuminuric DKD patients typically do not exhibit the usual signs of proteinuria or renal dysfunction but still face a higher cardiovascular risk (9). Given the metabolic abnormalities and early kidney damage in these patients, personalized treatment strategies are crucial. Dyslipidemia plays a significant role in DKD progression, so clinicians should tailor treatment plans based on individual factors, including glycemic control, lipid levels, renal function, and other cardiovascular risk factors. It is important to avoid medications that may further burden the kidneys and to consider nephroprotective drugs, such as SGLT2 inhibitors and GLP-1 receptor agonists. Understanding the differences between these two phenotypes could help develop more targeted therapeutic strategies and improve risk stratification in DKD management.

Given the significant heterogeneity in lipid profiles and metabolic responses among DKD patients, precision medicine strategies for individualized lipid management have emerged as a key area for future research. Different patients may exhibit distinct lipid metabolism abnormalities, such as elevated triglycerides (TG), low high-density lipoprotein cholesterol (HDL-C), or small, dense low-density lipoprotein (sd-LDL), each of which may contribute differently to DKD progression. A one-size-fits-all lipid-lowering approach may not be optimal; instead, accurately identifying a patient’s lipid profile and tailoring treatments based on specific pathophysiological mechanisms could help enhance therapeutic efficacy, minimize adverse effects, and improve long-term DKD outcomes. Future studies should explore differential responses to lipid-lowering therapies among DKD subtypes and integrate genomics, metabolomics, and biomarker-based screening to develop more targeted and personalized treatment strategies.

The emerging understanding of dyslipidemia’s intricate role in the pathogenesis of DKD underscores the need for continued exploration and innovation in therapeutic approaches. Future developments in the management of dyslipidemia in DKD are likely to revolve around precision medicine approaches, novel therapeutic targets, combination therapies, non-pharmacological interventions, and advanced therapeutic modalities. Continued interdisciplinary collaboration between researchers, clinicians, and industry stakeholders will be essential in translating these insights into meaningful improvements in clinical outcomes for patients with DKD.

## References

[1] de Boer IH Khunti K Sadusky T Tuttle KR Neumiller JJ Rhee CM. Diabetes management in chronic kidney disease: a consensus report by the American Diabetes Association (ADA) and Kidney Disease: Improving Global Outcomes (KDIGO). Kidney Int. (2022) 102:974–89. doi: 10.1016/j.kint.2022.08.012 36202661

[2] Duan JY Duan GC Wang CJ Liu DW Qiao YJ Pan SK. Prevalence and risk factors of chronic kidney disease and diabetic kidney disease in a central Chinese urban population: a cross-sectional survey. BMC Nephrol. (2020) 21:115. doi: 10.1186/s12882-020-01761-5 32245423 PMC7118942

[3] Gupta S Dominguez M Golestaneh L. Diabetic kidney disease: an update. Med Clin North Am. (2023) 107:689–705. doi: 10.1016/j.mcna.2023.03.004 37258007

[4] Kaze AD Santhanam P Erqou S Bertoni AG Ahima RS Echouffo-Tcheugui JB. Microvascular disease and cardiovascular outcomes among individuals with type 2 diabetes. Diabetes Res Clin Pract. (2021) 176:108859. doi: 10.1016/j.diabres.2021.108859 33989668 PMC8627586

[5] González-Pérez A Saez M Vizcaya D Lind M Garcia Rodriguez L. Incidence and risk factors for mortality and end-stage renal disease in people with type 2 diabetes and diabetic kidney disease: a population-based cohort study in the UK. BMJ Open Diabetes Res Care. (2021) 9:e002146. doi: 10.1136/bmjdrc-2021-002146 PMC8491294 34607828

[6] Ninomiya T Perkovic V de Galan BE Zoungas S Pillai A Jardine M. Albuminuria and kidney function independently predict cardiovascular and renal outcomes in diabetes. J Am Soc Nephrol. (2009) 20:1813–21. doi: 10.1681/ASN.2008121270 PMC2723977 19443635

[7] Svensson MK Cederholm J Eliasson B Zethelius B Gudbjörnsdottir S Swedish National Diabetes Register. Albuminuria and renal function as predictors of cardiovascular events and mortality in a general population of patients with type 2 diabetes: a nationwide observational study from the Swedish National Diabetes Register. Diabetes Vasc Dis Res. (2013) 10:520–9. doi: 10.1177/1479164113500798 24002670

[8] Di Pino A Scicali R Marchisello S Zanoli L Ferrara V Urbano F. High glomerular filtration rate is associated with impaired arterial stiffness and subendocardial viability ratio in prediabetic subjects. Nutr Metab Cardiovasc Dis. (2021) 31:3393–400. doi: 10.1016/j.numecd.2021.08.030 34625357

[9] Di Marco M Scilletta S Miano N Marrano N Natalicchio A Giorgino F. Cardiovascular risk and renal injury profile in subjects with type 2 diabetes and non-albuminuric diabetic kidney disease. Cardiovasc Diabetol. (2023) 22:344. doi: 10.1186/s12933-023-02065-2 38093293 PMC10720121

[10] Farah RI Al-Sabbagh MQ Momani MS Albtoosh A Arabiat M Abdulraheem AM. Diabetic kidney disease in patients with type 2 diabetes mellitus: a cross-sectional study. BMC Nephrol. (2021) 22:223. doi: 10.1186/s12882-021-02429-4 34134654 PMC8207700

[11] Gong L Wang C Ning G Wang W Chen G Wan Q. High concentrations of triglycerides are associated with diabetic kidney disease in new-onset type 2 diabetes in China: Findings from the China Cardiometabolic Disease and Cancer Cohort (4C) Study. Diabetes Obes Metab. (2021) 23:2551–60. doi: 10.1111/dom.v23.11 PMC9291490 34322974

[12] Matsuoka-Uchiyama N Uchida HA Okamoto S Onishi Y Katayama K Tsuchida-Nishiwaki M. The association of postprandial triglyceride variability with renal dysfunction and microalbuminuria in patients with type 2 diabetic mellitus: A retrospective and observational study. J Diabetes Res. (2022) 2022:3157841. doi: 10.1155/2022/3157841 35047644 PMC8763569

[13] Russo G Piscitelli P Giandalia A Viazzi F Pontremoli R Fioretto P. Atherogenic dyslipidemia and diabetic nephropathy. J Nephrol. (2020) 33:1001–8. doi: 10.1007/s40620-020-00739-8 32328901

[14] Russo GT De Cosmo S Viazzi F Pacilli A Ceriello A Genovese S. Plasma triglycerides and HDL-C levels predict the development of diabetic kidney disease in subjects with type 2 diabetes: the AMD annals initiative. Diabetes Care. (2016) 39:2278–87. doi: 10.2337/dc16-1246 27703024

[15] Hirano T Satoh N Kodera R Hirashima T Suzuki N Aoki E. Dyslipidemia in diabetic kidney disease classified by proteinuria and renal dysfunction: A cross-sectional study from a regional diabetes cohort. J Diabetes Investig. (2022) 13:657–67. doi: 10.1111/jdi.13697 PMC9017612 34665936

[16] Hirano T. Pathophysiology of diabetic dyslipidemia. J Atheroscler Thromb. (2018) 25:771–82. doi: 10.5551/jat.RV17023 PMC6143775 29998913

[17] Sacks FM Hermans MP Fioretto P Valensi P Davis T Horton E. Association between plasma triglycerides and high-density lipoprotein cholesterol and microvascular kidney disease and retinopathy in type 2 diabetes mellitus: a global case-control study in 13 countries. Circulation. (2014) 129:999–1008. doi: 10.1161/CIRCULATIONAHA.113.002529 24352521

[18] Wang C Wang L Liang K Yan F Hou X Liu F. Poor control of plasma triglycerides is associated with early decline of estimated glomerular filtration rates in new-onset type 2 diabetes in China: results from a 3-year follow-up study. J Diabetes Res. (2020) 2020:3613041. doi: 10.1155/2020/3613041 33062710 PMC7542506

[19] Pontremoli R Desideri G Arca M Temporelli PL Perrone V Dovizio M. Hypertriglyceridemia is associated with decline of estimated glomerular filtration rate and risk of end-stage kidney disease in a real-word Italian cohort: Evidence from the TG-RENAL Study. Eur J Intern Med. (2023) 111:90–6. doi: 10.1016/j.ejim.2023.02.019 36906475

[20] Bjornstad P Maahs DM Wadwa RP Pyle L Rewers M Eckel RH. Plasma triglycerides predict incident albuminuria and progression of coronary artery calcification in adults with type 1 diabetes: the Coronary Artery Calcification in Type 1 Diabetes Study. J Clin Lipidol. (2014) 8:576–83. doi: 10.1016/j.jacl.2014.08.008 PMC4268486 25499940

[21] Yang H Young D Gao J Yuan Y Shen M Zhang Y. Are blood lipids associated with microvascular complications among type 2 diabetes mellitus patients? A cross-sectional study in Shanghai, China. Lipids Health Dis. (2019) 18:18. doi: 10.1186/s12944-019-0970-2 30658647 PMC6339385

[22] Yun KJ Kim HJ Kim MK Kwon HS Baek KH Roh YJ. Risk factors for the development and progression of diabetic kidney disease in patients with type 2 diabetes mellitus and advanced diabetic retinopathy. Diabetes Metab J. (2016) 40:473–81. doi: 10.4093/dmj.2016.40.6.473 PMC5167712 27766790

[23] Uemura T Nishimoto M Eriguchi M Tamaki H Tasaki H Furuyama R. Association of triglycerides to high-density lipoprotein cholesterol ratio with incident cardiovascular disease but not end-stage kidney disease among patients with biopsy-proven diabetic nephropathy. Hypertens Res. (2023) 46:1423–32. doi: 10.1038/s41440-023-01197-y 36750609

[24] Oliveira HCF Raposo HF. Cholesteryl ester transfer protein and lipid metabolism and cardiovascular diseases. Adv Exp Med Biol. (2020) 1276:15–25. doi: 10.1007/978-981-15-6082-8_2 32705591

[25] Opazo-Ríos L Mas S Marín-Royo G Mezzano S Gómez-Guerrero C Moreno JA. Lipotoxicity and diabetic nephropathy: novel mechanistic insights and therapeutic opportunities. Int J Mol Sci. (2020) 21:2632. doi: 10.3390/ijms21072632 32290082 PMC7177360

[26] Jang HS Noh MR Kim J Padanilam BJ. Defective mitochondrial fatty acid oxidation and lipotoxicity in kidney diseases. Front Med (Lausanne). (2020) 7:65. doi: 10.3389/fmed.2020.00065 32226789 PMC7080698

[27] Sun Y Cui S Hou Y Yi F. The updates of podocyte lipid metabolism in proteinuric kidney disease. Kidney Dis (Basel). (2021) 7:438–51. doi: 10.1159/000518132 PMC8613610 34901191

[28] Herman-Edelstein M Scherzer P Tobar A Levi M Gafter U. Altered renal lipid metabolism and renal lipid accumulation in human diabetic nephropathy. J Lipid Res. (2014) 55:561–72. doi: 10.1194/jlr.P040501 PMC3934740 24371263

[29] Roumeliotis S Roumeliotis A Georgianos PI Stamou A Manolopoulos VG Panagoutsos S. Oxidized LDL is associated with eGFR decline in proteinuric diabetic kidney disease: A cohort study. Oxid Med Cell Longev. (2021) 2021:2968869. doi: 10.1155/2021/2968869 34712380 PMC8548137

[30] Li R Shi T Xing E Qu H. Atorvastatin calcium tablets on inflammatory factors, hemorheology and renal function damage indexes in patients with diabetic nephropathy. Pak J Med Sci. (2021) 37:1392–6. doi: 10.12669/pjms.37.5.4045 PMC8377901 34475918

[31] Shen X Zhang Z Zhang X Zhao J Zhou X Xu Q. Efficacy of statins in patients with diabetic nephropathy: a meta-analysis of randomized controlled trials. Lipids Health Dis. (2016) 15:179. doi: 10.1186/s12944-016-0350-0 27733168 PMC5062823

[32] Qin X Dong H Fang K Lu F. The effect of statins on renal outcomes in patients with diabetic kidney disease: A systematic review and meta-analysis. Diabetes Metab Res Rev. (2017) 33. doi: 10.1002/dmrr.v33.6 28477396

[33] Lv J Ren C Hu Q. Effect of statins on the treatment of early diabetic nephropathy: a systematic review and meta-analysis of nine randomized controlled trials. Ann Palliat Med. (2021) 10:11548–57. doi: 10.21037/apm-21-2673 34872280

[34] Hanai K Babazono T Uchigata Y. Effects of statins on the kidneys in patients with type 2 diabetes. Clin Exp Nephrol. (2017) 21:633–42. doi: 10.1007/s10157-016-1329-x 27631405

[35] Huang TS Wu T Wu YD Li XH Tan J Shen CH. Long-term statins administration exacerbates diabetic nephropathy via ectopic fat deposition in diabetic mice. Nat Commun. (2023) 14:390. doi: 10.1038/s41467-023-35944-z 36693830 PMC9873739

[36] Sanvee GM Panajatovic M Bouitbir DJ Krähenbühl D. Statins and insulin resistance. Eur Cardiol. (2020) 15:e44. doi: 10.15420/ecr.2020.15.1.PO21 32612704 PMC7312546

[37] Jairoun AA Ping CC Ibrahim B. Statin therapy for patients with diabetic nephropathy: balance between safety and efficacy of statin treatment for patients with impaired kidney function. Eur Rev Med Pharmacol Sci. (2023) 27:10595–604. doi: 10.26355/eurrev_202311_34339 37975384

[38] Frazier R Mehta R Cai X Lee J Napoli S Craven T. Associations of fenofibrate therapy with incidence and progression of CKD in patients with type 2 diabetes. Kidney Int Rep. (2018) 4:94–102. doi: 10.1016/j.ekir.2018.09.006 30596172 PMC6308372

[39] Davis TM Ting R Best JD Donoghoe MW Drury PL Sullivan DR. Effects of fenofibrate on renal function in patients with type 2 diabetes mellitus: the Fenofibrate Intervention and Event Lowering in Diabetes (FIELD) Study. Diabetologia. (2011) 54:280–90. doi: 10.1007/s00125-010-1951-1 21052978

[40] Ting RD Keech AC Drury PL Donoghoe MW Hedley J Jenkins AJ. Benefits and safety of long-term fenofibrate therapy in people with type 2 diabetes and renal impairment: the FIELD Study. Diabetes Care. (2012) 35:218–25. doi: 10.2337/dc11-1109 PMC3263870 22210576

[41] Yen CL Fan PC Lin MS Lee CC Tu KH Chen CY. Fenofibrate delays the need for dialysis and reduces cardiovascular risk among patients with advanced CKD. J Clin Endocrinol Metab. (2021) 106:1594–605. doi: 10.1210/clinem/dgab137 33677489

[42] Tang C Deng X Qu J Miao Y Tian L Zhang M. Fenofibrate attenuates renal tubular cell apoptosis by up-regulating MCAD in diabetic kidney disease. Drug Des Devel Ther. (2023) 17:1503–14. doi: 10.2147/DDDT.S405266 PMC10202114 37223723

[43] Horinouchi Y Murashima Y Yamada Y Yoshioka S Fukushima K Kure T. Pemafibrate inhibited renal dysfunction and fibrosis in a mouse model of adenine-induced chronic kidney disease. Life Sci. (2023) 321:121590. doi: 10.1016/j.lfs.2023.121590 36940907

[44] Kito K Nomoto H Sakuma I Nakamura A Cho KY Kameda H. Effects of pemafibrate on lipid metabolism in patients with type 2 diabetes and hypertriglyceridemia: A multi-center prospective observational study, the PARM-T2D study. Diabetes Res Clin Pract. (2022) 192:110091. doi: 10.1016/j.diabres.2022.110091 36174777

[45] Das Pradhan A Glynn RJ Fruchart JC MacFadyen JG Zaharris ES Everett BM. Triglyceride lowering with pemafibrate to reduce cardiovascular risk. N Engl J Med. (2022) 387:1923–34. doi: 10.1056/NEJMoa2210645 36342113

[46] Seki M Nakano T Tanaka S Matsukuma Y Funakoshi K Ohkuma T. Design and methods of an open-label, randomized controlled trial to evaluate the effect of pemafibrate on proteinuria in CKD patients (PROFIT-CKD). Clin Exp Nephrol. (2023) 27:358–64. doi: 10.1007/s10157-023-02322-4 36738362

[47] Bruce R Daniels A Cundy T. Renal function changes in diabetic nephropathy induced by bezafibrate. Nephron. (1996) 73:490. doi: 10.1159/000189120 8832617

[48] Ansquer JC Foucher C Rattier S Taskinen MR Steiner G DAIS Investigators. Fenofibrate reduces progression to microalbuminuria over 3 years in a placebo-controlled study in type 2 diabetes: results from the Diabetes Atherosclerosis Intervention Study (DAIS). Am J Kidney Dis. (2005) 45:485–93. doi: 10.1053/j.ajkd.2004.11.004 15754270

[49] Sun X Liu J Wang G. Fenofibrate decreased microalbuminuria in the type 2 diabetes patients with hypertriglyceridemia. Lipids Health Dis. (2020) 19:103. doi: 10.1186/s12944-020-01254-2 32446306 PMC7245839

[50] Yanai H Katsuyama H Hakoshima M. A significant increase of estimated glomerular filtration rate after switching from fenofibrate to pemafibrate in type 2 diabetic patients. Cardiol Res. (2021) 12:358–62. doi: 10.14740/cr1333 PMC8683099 34970366

[51] Imbalzano E Ilardi F Orlando L Pintaudi B Savarese G Rosano G. The efficacy of PCSK9 inhibitors on major cardiovascular events and lipid profile in patients with diabetes: a systematic review and meta-analysis of randomized controlled trials. Eur Heart J Cardiovasc Pharmacother. (2023) 9:318–27. doi: 10.1093/ehjcvp/pvad019 36972610

[52] Feng Z Liao X Peng J Quan J Zhang H Huang Z. PCSK9 causes inflammation and cGAS/STING pathway activation in diabetic nephropathy. FASEB J. (2023) 37:e23127. doi: 10.1096/fj.202300342RRR 37561547

[53] Feng Z Liao X Zhang H Peng J Huang Z Yi B. Increased serum PCSK9 levels are associated with renal function impairment in patients with type 2 diabetes mellitus. Ren Fail. (2023) 45:2215880. doi: 10.1080/0886022X.2023.2215880 37246753 PMC10228320

[54] Elewa U Fernández-Fernández B Mahillo-Fernández I Martin-Cleary C Sanz AB Sanchez-Niño MD. PCSK9 in diabetic kidney disease. Eur J Clin Invest. (2016) 46:779–86. doi: 10.1111/eci.2016.46.issue-9 27438893

[55] Cowart K Singleton J Carris NW. Inclisiran for the treatment of hyperlipidemia and for atherosclerotic cardiovascular disease risk reduction: A narrative review. Clin Ther. (2023) 45(11):1099–104. doi: 10.1016/j.clinthera.2023.06.011 37451914

[56] Di-Giacomo-Barbagallo F Andreychuk N Scicali R Gonzalez-Lleó A Piro S Masana L. Inclisiran, reasons for a novel agent in a crowded therapeutic field. Curr Atheroscler Rep. (2025) 27:25. doi: 10.1007/s11883-024-01271-x 39786678 PMC11717820

[57] Ray KK Troquay RPT Visseren FLJ Leiter LA Scott Wright R Vikarunnessa S. Long-term efficacy and safety of inclisiran in patients with high cardiovascular risk and elevated LDL cholesterol (ORION-3): results from the 4-year open-label extension of the ORION-1 trial. Lancet Diabetes Endocrinol. (2023) 11:109–19. doi: 10.1016/S2213-8587(22)00353-9 36620965

[58] Ray KK Raal FJ Kallend DG Jaros MJ Koenig W Leiter LA. Inclisiran and cardiovascular events: a patient-level analysis of phase III trials. Eur Heart J. (2023) 44:129–38. doi: 10.1093/eurheartj/ehac594 PMC9825807 36331326

[59] Wada T Hara A Muso E Maruyama S Kato S Furuichi K,Y. Effects of LDL apheresis on proteinuria in patients with diabetes mellitus, severe proteinuria, and dyslipidemia. Clin Exp Nephrol. (2021) 25:1–8. doi: 10.1007/s10157-020-01959-9 32857255

[60] Hara A Wada T Muso E Maruyama S Kato S Furuichi K. Effect of low-density lipoprotein apheresis on quality of life in patients with diabetes, proteinuria, and hypercholesterolemia. Blood Purif. (2023) 52:373–81. doi: 10.1159/000527900 36521435

[61] Ito H Naito C Hayashi H Kawamura M Miyazaki S. Selective removal of triglyceride-rich lipoproteins by plasmapheresis in diabetic patients with severe hypertriglyceridemia. Artif Organs. (1989) 13:190–6. doi: 10.1111/j.1525-1594.1989.tb02862.x 2764758

[62] Liu WS Lin CH Tsai CY Wang HT Li SY Liu TY. Double filtration plasmapheresis with polyvinyl alcohol-based membrane lowers serum inflammation and toxins in patients with hyperlipidemia. Bioengineering (Basel). (2023) 10:89. doi: 10.3390/bioengineering10010089 36671661 PMC9855020

[63] Zhang XM Gu YH Deng H Xu ZQ Zhong ZY Lyu XJ. Plasma purification treatment relieves the damage of hyperlipidemia to PBMCs. Front Cardiovasc Med. (2021) 8:691336. doi: 10.3389/fcvm.2021.691336 34307504 PMC8292646

[64] Scilletta S Di Marco M Miano N Filippello A Di Mauro S Scamporrino A. Update on diabetic kidney disease (DKD): focus on non-albuminuric DKD and cardiovascular risk. Biomolecules. (2023) 13:752. doi: 10.3390/biom13050752 37238622 PMC10216614

